# Boredom Proneness and Online Deviant Behaviors: The Mediating Role of Rumination and the Moderating Role of Gender

**DOI:** 10.3390/bs12110455

**Published:** 2022-11-16

**Authors:** Jing Zhao, Shisheng Chen, Xiaochun Xie, Jin Wang, Xiaodong Wang

**Affiliations:** 1 School of Humanities and Social Sciences, North University of China, Taiyuan 030051, China; 2 Department of Student Affairs, Fujian College of Water Conservancy and Electric Power, Yongan 366000, China; 3 School of Psychology, Northeast Normal University, Changchun 130024, China; 4 School of Mechanical Engineering, North University of China, Taiyuan 030051, China

**Keywords:** online deviant behaviors, boredom proneness, rumination, gender

## Abstract

Online deviant behaviors have received increasing attention. This study examined the association between boredom proneness and online deviant behaviors as well as the mediating role of rumination and the moderating role of gender in the relationship. A sample of 1001 college students (Mage = 20.20 ± 1.52 years, 50.25% female) was recruited to complete a set of questionnaires assessing the main variables. The results show that boredom proneness was positively associated with online deviant behaviors and that rumination played a mediating role in this relationship. Moreover, gender differences were found in the relationship, which was stronger for males than females. Despite several limitations, this study deepens our understanding of the influencing mechanism of boredom proneness on online deviant behaviors, which could provide practical implications for the prevention and intervention of online deviant behaviors.

## 1. Introduction

With the development of information technology, the Internet has played an important role in people’s lives. Especially during the COVID-19 pandemic, people have had to rely more on the Internet to maintain social contacts, work, and study because of family isolation. Compared to the end of 2019, China’s Internet traffic had increased by approximately 50% by mid-2020 [1]. However, the ever-increasing rate of Internet use is a double-edged sword that has brought convenience to our lives and is inevitably accompanied by deviant behaviors. Most notably, 34.5% of Chinese juvenile Internet users have encountered various kinds of undesirable Internet information, such as obscenity, bloody violence, self-mutilation, and suicide [2]. Therefore, online deviant behaviors have received increasing attention from researchers.

In the literature, online deviant behaviors usually refer to cyber delinquency, cyber deviance, or online deviance [3,4,5]. These are types of behaviors that refer to harming oneself or others because the individual is not adjusting well to the Internet environment through online flaming, deception on the Internet, and online obscenity and pornography [6,7,8,9]. Online deviant behaviors are closely related to academic failure, psychological crises, and criminal behaviors [7,10,11]. Given these adverse effects of online deviant behaviors, it is necessary to identify trigger factors and underlying mechanisms.

Previous studies have shown that individual factors (such as moral disengagement [12], self-control [13], interpersonal needs [14], etc.) and environmental factors (for example, Internet anonymity [13], social ostracism [15], family patterns [16], peer network deviant behaviors [17], etc.) are closely related to online deviant behaviors. However, less is known about the psychopathology-related variables among individual factors. Boredom is ubiquitous in human existence [18]; especially during the COVID-19 pandemic, boredom was reported as one of the most salient negative experiences [19]. Therefore, we explored the association between boredom and online deviant behaviors.

### 1.1. Boredom Proneness and Online Deviant Behaviors

In previous studies, there have been two main aspects of boredom: state boredom and trait boredom. When boredom is experienced as a result of external circumstances, it is called state boredom, which is situation-dependent and transient [20]. State boredom is not intrinsically harmful but how a person responds to boredom can lead to either positive or negative consequences [21]. Additionally, the different ways to cope with boredom might depend in part on individual differences in boredom proneness. Boredom proneness is viewed as a trait, which affects an individual’s perception of environmental stimulation and persists through situational change [20,22]. Individuals with high boredom proneness are more likely to involve attentional and impulse control difficulties, which leads to momentary boredom and thus the negative consequences [23]. Following this reason, the current study focuses on boredom proneness.

According to sensation-seeking theory and arousal theory, people who maintain their health must be exposed to a variety of stimuli to achieve optimal arousal levels [22,24]. However, individuals with a high level of boredom proneness are more likely to perceive the environment as monotonous and constrained; thus, they would have a strong desire for sensation seeking, such as substances use [25], alcohol abuse [26], rule breaking [27], social network addiction [28], and problematic smartphone use [29]. Boredom proneness is a prominent risk factor for deviant behaviors. A study has confirmed that boredom proneness and online deviant behaviors are significantly correlated [30].

### 1.2. The Mediation of Rumination

Recently, rumination—one’s tendency to think repetitively, uncontrollably, and intrusively about the possible causes and consequences of stressors [31]—has received growing attention as a risk factor for deviant behavior. It is regarded as a highly dysfunctional cognitive strategy for coping with stressful events [32]. Research has shown that rumination positively correlates with offline passive consequences (such as depression [33,34], aggression [35], suicide [36], and so on), and online negative outcomes (such as problematic mobile phone use [37], online trolling [38], and so on). Resource depletion theory argues that rumination leads to individuals’ limited cognitive resources being occupied too much and results in damaged executive control function and failure of self-control [39]; thus, individuals with rumination are prone to engage in deviant behaviors. Consequently, we deem that rumination is positively associated with online deviant behaviors.

According to the stress-reactive model of rumination, individuals who experience a stressful event or negative emotion would experience rumination [32]. As a common negative emotion, boredom positively correlates with rumination [40,41,42]. Similarly, elaborated control theory may explain this relationship; that is, rumination occurs when people recognize discrepancies between desired goals and current states [43]. In addition, boredom reflects a discrepancy between the current, meaningless situation and a desired, more meaningful situation [44]. However, these studies mainly focused on boredom in certain situations (for example, workplace, school, during the COVID-19 lockdown, etc.). Whether one feels boredom may partly depend on boredom proneness and it is possible that individuals with high boredom proneness struggle with more feelings of boredom. Based on this reasoning, we deem that boredom proneness correlates with rumination.

Taken together, we put forward the hypothesis that rumination plays a mediating role between boredom proneness and online deviant behaviors.

### 1.3. The Moderation of Gender

Gender differences in online deviant behaviors have been examined in previous studies. Males are more likely to engage in online deviant behaviors than females [5,18], particularly in certain forms of online deviant behaviors (such as deviant cyber-sexual activities [45] and cyberbullying [46]). Hence, we consider gender differences here and deem that gender may act as a moderator between boredom proneness and online deviant behaviors. There are two reasons for this: First, according to the general strain theory, male with strains are more conducive to violence, while females are more prone to the escapist form of crime [47]. Being engaged in boredom is regarded as a strain; thus, males with high boredom proneness engage in more online deviant behaviors than females. Second, sensation-seeking theory confirms that someone with a high level of boredom proneness tends to engage in high sensation-seeking activities to avoid or reduce boredom and empirical studies have shown that males prefer exciting and risky behaviors compared to females, such as online deviant behaviors.

Furthermore, the stress-reactive model of rumination states that rumination can exaggerate the influence of extreme information on cognition, which makes it difficult for individuals to disengage from negative information [31]. Hence, rumination may aggravate the relationship between boredom and online deviant behaviors. Owing to gender differences in online deviant behaviors, we deem that gender also plays a moderating role between rumination and online deviant behaviors. That is, for males, rumination results in more online deviant behaviors than females.

Taken together, we suggest that gender plays a moderating role in boredom proneness, rumination, and online deviant behaviors. In particular, gender plays moderating in two paths: ”boredom proneness → online deviant behaviors” and “rumination → online deviant behaviors”. Males perform more online deviant behaviors than females.

## 2. Materials and Methods

### 2.1. Participants

A convenience sampling method was adopted to recruit students (including undergraduate and vocational college students) to participate in this study. After obtaining informed consent, a sample of 1100 participants anonymously completed an online questionnaire that could be completed in ten minutes. Because they reported the same values for all items, 99 participants were excluded. The remaining 1001 valid responses were used for further analysis. Among the total sample (*M*_age_ = 20.20, *SD*_age_ = 1.52, Range_age_ = 17–24), 518 were undergraduate students and 483 were vocational college students; 493 were male and 508 were female.

### 2.2. Measures

#### 2.2.1. Online Deviant Behaviors

The Scale of Adolescent Internet Deviance [48], a widely used scale in previous studies in China, was adopted to measure online deviant behaviors with 35 items. These items can be divided into three dimensions: online flaming (e.g., “when I have a conflict with someone online, I will send them offensive symbols/pictures”), online cheating behaviors (e.g., “I often make up my own experience”), and online pornography (e.g., “On the Internet, I download/watch pornographic movies/pictures”). Participants were asked to respond on a five-point scale (1 “never” to 5 ”always”), with higher scores indicating a higher frequency of online deviant behaviors. Cronbach’s alpha for this scale was 0.90 in this study.

#### 2.2.2. Boredom Proneness

The Short Boredom Proneness Scale developed by Struk et al. with eight items (e.g., “I often find myself at ‘loose ends,’ not knowing what to do.”) was adopted, which has been translated and used in Chinese studies with adequate validity and reliability [49,50]. Participants were asked to respond to each item on a seven-point scale ranging from 1 “Strongly Disagree” to 7 “Strongly Agree”. A higher score indicates a higher level of boredom and Cronbach’s alpha for this scale was 0.86 in this study.

#### 2.2.3. Rumination

Nolen-Hoeksema Ruminative Responses Scale, translated and used in Chinese samples with favorable validity and reliability, was adopted to measure rumination with 22 items [51]. Participants were asked to respond to each item on a four-point scale (1 “never” to 4 “always”) and a higher score indicates a higher level of ruminative responses. Cronbach’s alpha for this scale was 0.87 in this study.

### 2.3. Data Analysis

All statistical analyses were conducted with SPSS 25.0. First, descriptive statistics and correlational analyses were conducted. Second, the PROCESS macro for SPSS was adopted to test the moderated mediation model with 5000 bias-corrected samples, and the effect was considered significant when the 95% confidence interval (CI) did not include zero [52]. Specifically, Model 4 was used to test the mediating model with rumination as the mediator; Model 15 was used to test the integrated model with rumination as the mediator and gender as the moderator.

## 3. Results

### 3.1. Test for Common Method Bias

Using self-reported questionnaires to collect data may lead to common method bias. To reduce this possible bias, we used some methods, such as anonymous surveys, appropriate changes in response sentences (such as strongly disagree or strongly agree, never or always), and different scoring methods (such as four points, five points, and seven points). Statistical analyses were performed using Harman’s single-factor test. The results show that there were seven factors with a characteristic root greater than 1 and the first factor explaining the cumulative variation is 38.77%, which is less than 40%, indicating that there is not serious problem with common method bias [53].

### 3.2. Descriptive Statistics and Correlations between Main Variables

Table 1 presents the means, standard deviations, and Pearson’s correlations among the main variables. Boredom proneness was positively correlated with rumination and online deviant behaviors and rumination was positively correlated with online deviant behaviors.

### 3.3. Testing the Hypothesized Moderated Mediation Model

The PROCESS macro for SPSS with 5000 bootstrapping samplings was used to test the proposed hypotheses [54]. First, the simple mediating model analysis (Model 4) is shown in Table 2. The total effect of boredom proneness on online deviant behaviors was 0.36 (Boot SE = 0.03; Boot 95% CI = (0.30; 0.42)) and the mediating effect of rumination was 0.17 (Boot SE = 0.02; Boot 95% CI = (0.12; 0.22)), which accounted for 46.34% of the total effect.

**Figure 1:**
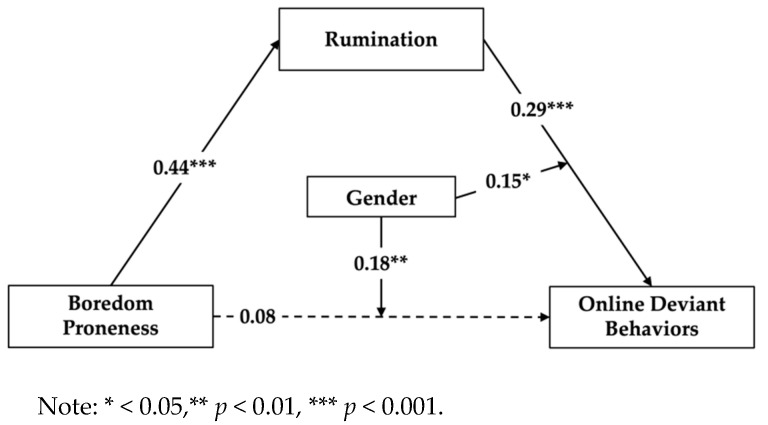
The moderated mediation model.

Second, the PROCESS macro for SPSS (Model 15) was used to examine the moderated mediation model (shown in Figure 1). The main results consist of two parts: the regression analysis model and conditional effect analysis and they are presented in Table 2 and Table 3, respectively. As shown in Table 2, boredom proneness was positively associated with rumination and rumination was positively associated with online deviant behaviors, while boredom proneness was not significantly associated with online deviant behaviors. Therefore, rumination can fully mediate the association between boredom proneness and online deviant behaviors. Moreover, the interaction effects of boredom proneness and gender, rumination, and gender on online deviant behaviors were significant, indicating gender played the moderating role in the association between boredom, rumination, and online deviant behaviors.

Finally, as shown in Table 3, the direct effects of boredom proneness differed between males and females. For females, the direct effect included zero; for males, the direct effect was positive and excluded zero. For males and females, the mediating effects were positive and excluded zero and the moderated mediation index was significant. That is, for males, the mediating effect of rumination was significantly stronger than for females.

**Figure 2:**
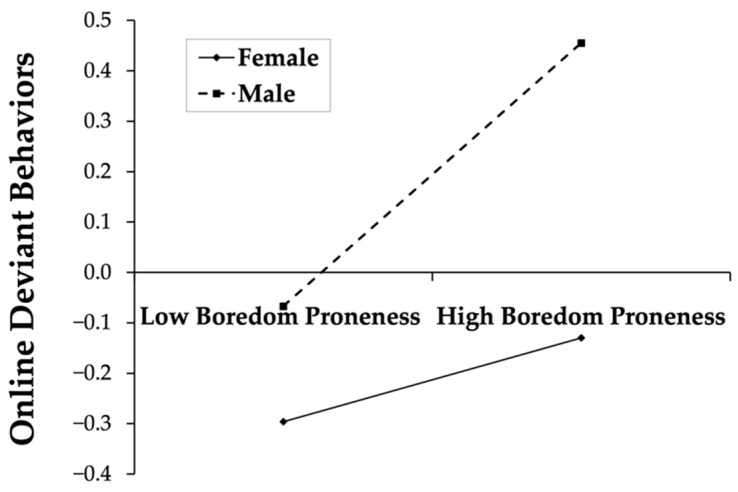
The association between boredom proneness and online deviant behaviors for gender.

**Figure 3:**
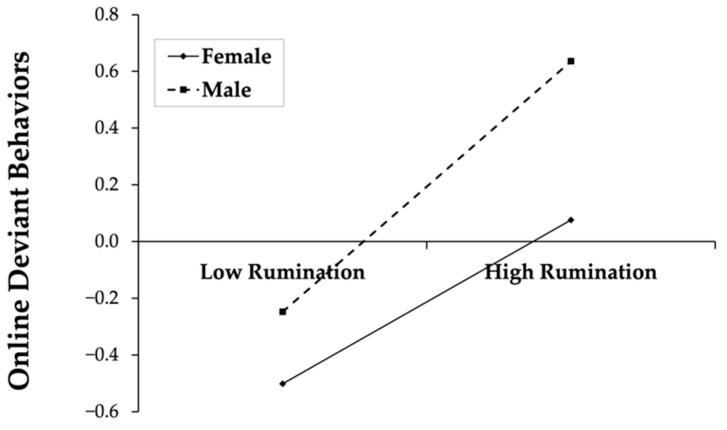
The association between rumination and online deviant behaviors for gender.

To examine the moderation of gender, simple slope tests were performed and simple effect analysis plots were drawn (Figure 2 and Figure 3). The results shown in Figure 2 demonstrate that, for females, there was no significant correlation between boredom proneness and online deviant behaviors (simple slope = 0.08, t = 1.90, *p* > 0.05); for males, boredom proneness is significantly correlated with online deviant behaviors (simple slope = 0.26, t = 6.52, *p* < 0.01); in other words, males with higher levels of boredom proneness may exhibit more online deviant behaviors. As shown in Figure 3, for females, the positive correlation between rumination and online deviant behaviors was significant (simple slope = 0.29, t = 6.46, *p* < 0.01); while for males, rumination also positively predicted online deviant behaviors (simple slope = 0.44, t = 5.91, *p* < 0.01).

## 4. Discussion

This study examined the association between boredom proneness and online deviant behaviors and the underlying mechanism. The results indicate that the association between boredom proneness and online deviant behaviors was stronger for males than females. For males, boredom proneness is not only directly related to online deviant behaviors but also indirectly influenced online deviant behaviors through rumination; for females, boredom proneness was related to online deviant behaviors only through rumination.

### 4.1. Boredom Proneness and Online Deviant Behaviors

As hypothesized, the results suggest that boredom proneness is positively associated with online deviant behaviors. Consistent with research on other risky behaviors [24,25,26,27,28,29], boredom proneness is considered a risk factor for online deviant behaviors. In line with sensation-seeking theory and arousal theory, individuals with high boredom proneness are usually in a low-arousal state, which promotes them to engage in risky behaviors; moreover, their life satisfaction is lower, which urges them to turn to the Internet [54]. Thus, they may tend to exhibit online deviant behaviors to achieve an optimal arousal state. In addition, dual self-consciousness theory can also explain this phenomenon. When individuals with high boredom proneness use the Internet, their private self-consciousness increases and public self-consciousness decreases [30]; thus, they pay more attention to their own feelings and less attention to others, causing individuals to engage in online deviant behaviors.

### 4.2. Rumination as a Mediator

Rumination mediated the positive association between boredom proneness and online deviant behaviors. As a kind of a predisposed vulnerability, individuals with boredom proneness are more likely to fall into the feelings of boredom, which is associated with emotional exhaustion [40]. According to the stress-reactive model of rumination, individuals with negative emotions are more likely to provoke rumination [32]. Therefore, individuals with high levels of boredom proneness are prone to trigger rumination.

On the other hand, rumination can increase online deviant behaviors. Rumination continuously directs individuals’ attention to boredom with a non-accepting attitude and prevents them from properly disengaging from boredom. That is, individuals engaged in rumination exaggerate the influence of boredom rather than actively taking action to get out of boredom; moreover, they become extremely sensitive to various negative stimuli. The Internet is full of various types of negative information; thus, when using the Internet, someone with rumination is more likely to engage in online deviant behaviors due to anonymity [38]. In addition, this study supports resource depletion theory [39], that is, when an individual’s cognitive resources are limited. Rumination induced by boredom leads to cognitive resources being more occupied and normal cognitive mechanisms being impaired; thus, they engage in more deviant behaviors when surfing the Internet. Therefore, rumination strengthens the association between boredom proneness and online deviant behaviors.

### 4.3. Gender as a Moderator

This study found different effects of boredom proneness on online deviant behaviors across gender. Specifically, among males, the total effect of boredom proneness on online deviant behaviors is 0.44, including the direct effect (0.26) and indirect effect through rumination (0.19); however, among females, the direct effect is not significant and the indirect effect through rumination is only 0.13. That is, males with boredom proneness are more likely to engage in online deviant behaviors. However, if rumination is not triggered, females with boredom proneness are less likely to engage in online deviant behaviors. This can be explained from several aspects. First, in line with general strain theory, males with boredom are more conducive to violence, while females are more prone to the escapist form of crime [47]; therefore, males with boredom proneness take more online deviant behaviors. Second, evolutionary psychology refers to the adaptive functions of violence, competition, and aggression for males, and gender differences in hormones (i.e., testosterone) provide a physiological basis for males’ deviant behaviors [55]. Third, from the perspective of gender socialization theory, females pay more attention to interpersonal relationships, whereas males are more likely to focus on competition and goal achievement due to gender roles and social norms [13]. Therefore, males with boredom proneness engage in more online deviant behaviors.

## 5. Implications and Limitations

The current findings have several implications. Theoretically, this study deepens our understanding of the risk factors and mechanisms of online deviant behaviors. Practically, the results may provide suggestions for the prevention and intervention of online deviant behaviors. First, given the high prevalence of boredom and the importance of boredom proneness in online deviant behaviors, mental health educators should pay more attention to students with high levels of boredom proneness and take active measures to raise their arousal state and reduce boredom experience, such as outdoor recreational activities. Second, rumination plays a mediating role between boredom proneness and online deviant behaviors and rumination strengthens their association. Thus, it is necessary to implement some interventions to reduce rumination, such as cognitive control training [56,57]. Third, the study indicated gender differences; therefore, mental health educators should focus on the online deviant behaviors of males with boredom proneness. While females with boredom proneness have less online deviant behaviors they may tend to internalize boredom elements. Therefore, mental health educators should encourage them to release boredom to avoid more serious psychological problems.

This study has several limitations. First, the self-reported method used may have led to deviations in this study; therefore, more objective measurements are needed. Second, causal inference cannot be achieved due to the cross-sectional design and a longitudinal or experimental design should be adopted in the future. Finally, participants were recruited from two universities in China, which may have limited the generalizability of the results. Future studies should recruit different categories of people so as to expand the generalizability.

## 6. Conclusions

We found a positive association between boredom proneness and online deviant behaviors as well as a mediating role of rumination and a moderating role of gender in this relationship. The results show that the association between boredom proneness and online deviant behaviors was stronger for males than for females. For males, boredom proneness is not only directly related to online deviant behaviors but also indirectly influences online deviant behaviors through rumination; for females, boredom proneness relates to online deviant behaviors only through rumination.

## Figures and Tables

**Table 1:** Means, standard deviations, and correlation results.

Variables	M(SD)	1	2	3	4
1 Gender	-	1			
2 Boredom Proneness	2.67 (1.50)	0.10 **	1		
3 Rumination	1.80 (0.60)	0.05	0.44 **	1	
4 Online Deviant behaviors	1.45 (0.64)	0.24 **	0.36 **	0.46 **	1

Note: ** *p* < 0.01; Gender—female “0”, male “1”.

**Table 2:** The regression analysis of the moderated mediation model.

Dependent Variable	Independent Variables	R^2^	F	β	Bootstrap LLCI	Bootstrap ULCI	t
Rumination	Boredom Proneness	0.19	241.91 ***	0.44	0.39	0.50	15.55 ***
Online Deviant behaviors (Model 4)	Boredom Proneness Rumination	0.24	159.72 ***	0.19	0.13	0.25	6.26 ***
				0.37	0.31	0.44	12.25 ***
Rumination	Boredom Proneness	0.19	241.91 ***	0.44	0.39	0.50	15.55 ***
Online Deviant behaviors	Boredom Proneness	0.30	86.36 ***	0.08	−0.002	0.17	1.91
(Model 15)	Rumination			0.29	0.20	0.37	6.33 ***
	Gender			0.41	0.30	0.51	7.65 ***
	Boredom Proneness × Gender			0.18	0.06	0.29	2.99 **
	Rumination × Gender			0.15	0.04	0.27	2.56 *

Note: * < 0.05, ** *p* < 0.01, *** *p* < 0.001. Gender: female “0”, male “1”; LL = low limit, CI = confidence interval, UL = upper limit.

**Table 3:** The conditional direct and indirect effect analysis.

Gender	Conditional Effect	Effect Value	Boot SE	Bootstrap LLCI	Bootstrap ULCI
Total	moderated mediation index	0.07	0.03	0.005	0.14
Male	Direct Effect	0.26	0.04	0.18	0.34
	Indirect Effect	0.19	0.03	0.14	0.26
Female	Direct Effect	0.08	0.04	−0.002	0.17
	Indirect Effect	0.13	0.02	0.09	0.17

Note: LL = low limit, CI = confidence interval, UL = upper limit.

## Data Availability

The data of this study are available from the corresponding author upon reasonable request.
